# Predicting Species Cover of Marine Macrophyte and Invertebrate Species Combining Hyperspectral Remote Sensing, Machine Learning and Regression Techniques

**DOI:** 10.1371/journal.pone.0063946

**Published:** 2013-06-03

**Authors:** Jonne Kotta, Tiit Kutser, Karolin Teeveer, Ele Vahtmäe, Merli Pärnoja

**Affiliations:** Estonian Marine Institute, University of Tartu, Tallinn, Estonia; The Australian National University, Australia

## Abstract

In order to understand biotic patterns and their changes in nature there is an obvious need for high-quality seamless measurements of such patterns. If remote sensing methods have been applied with reasonable success in terrestrial environment, their use in aquatic ecosystems still remained challenging. In the present study we combined hyperspectral remote sensing and boosted regression tree modelling (BTR), an ensemble method for statistical techniques and machine learning, in order to test their applicability in predicting macrophyte and invertebrate species cover in the optically complex seawater of the Baltic Sea. The BRT technique combined with remote sensing and traditional spatial modelling succeeded in identifying, constructing and testing functionality of abiotic environmental predictors on the coverage of benthic macrophyte and invertebrate species. Our models easily predicted a large quantity of macrophyte and invertebrate species cover and recaptured multitude of interactions between environment and biota indicating a strong potential of the method in the modelling of aquatic species in the large variety of ecosystems.

## Introduction

The ultimate goal in ecology is to understand biotic patterns and their changes in nature. In order to achieve an understanding, ecologists have spent much of their time engaged in mapping of different habitats or performing experiments to demonstrate interactions between physical environment and organisms. The vast majority of studies have been performed on limited spatial scales even though the studies covered larger areas than the grain size i.e. the size of sampling units still remained small and vast areas between grains were left unstudied [1], [2]. However, due to a wide range of spatial and complexity scales, the grain size profoundly affects how we see the world around us [3], [4]; hence flagging this issue as one of the critical problems in ecology [5].

There is an obvious need for high-quality mesoscale or even larger-scale spatially continuous measurements of biotic patterns either for validating current theories or to build better predictive spatial models. In recent decades there have been concomitant technological advances on the spatially continuous large-scale mapping of many of the Earth's habitats. Such remote sensing methods usually acquire information about an object or phenomenon over vast areas with 1 m or even higher spatial resolution. The remote sensing methods have been applied with reasonable success in terrestrial environment [6]. However, their use in aquatic ecosystems remains challenging because water is a strongly absorbing medium and the sensors used in remote sensing over water therefore must be very sensitive. Also, the strong attenuation of light by water and it's constituents limits the depth where any information can be collected and dampens the specific optical features that can be used to separate between different biotic features.

Currently it is believed that remote sensing does not allow mapping of aquatic communities at species level, except in simple environments with a few optically distinct species. The entire argument is based on the assumption that variability in optical signatures within species is far smaller than between species variability and earlier studies tried to classify the species accordingly. However, this is not likely in nature providing the complexity of fine-scale patterns of species distribution [7]. For example, there are coral species that vary in optical properties to the extent equal to the spectral variability of all corals [8]. The optical properties of green macroalgae and higher plants including seagrasses [9] are nearly identical, especially if the spectral resolution of the sensor used is not very fine. Although spectral unmixing methods have been proposed [10], the measured signal is usually an inseparable combination of signals from optically different objects [11].

Marine macrovegetation plays an irreplaceable role in maintaining coastal life by providing habitat as well as a source of organic matter and energy for upper trophic levels [12], [13]. Some plants such as seagrasses typically grow in monospecific stands but others may form mixed assemblages with varying amount of green, brown and red algae either attached on primary substrate or growing epiphytically on other algae. Similarly, seafloor may be covered either with small algal patches or lush benthic vegetation [14]. As the optical signature is formed when integrating information from spatial resolutions of meters to tens of metres, changes in spatial arrangement and densities of macrovegetation have a strong effect on the outcome [11], [15]. This leads to the conclusion that the optical signature may capture well algal cover but not necessarily its identity unless providing information on the algal cover. The distribution of assemblages is often characterised by a clear gradual continuum of changes in species densities and includes few sharp borders between classes [16]. Thus, any classification system tends to over-simplify natural assemblages whereas models incorporating species cover may succeed in replicating the species patterns.

The rising interest in marine habitat mapping has resulted in numerous modelling studies focussed on the distribution of species and habitats. Recently, generalized linear models enabled pioneering regression-based species distribution models. By handling non-normal error distributions, additive terms and nonlinear fitted function they provided useful flexibility for reproducing ecologically realistic relationships [17]. Moreover, the development of geographic information systems enabled the increasing range of emerging technologies to measure and share environmental data [18]. However, marine and freshwater applications are still rare [19], [20], [21], [22] compared to terrestrial modelling and these models are still based on surprisingly weak theoretical foundations [23]. This is because in the species distribution modelling, predictive purposes are usually aimed [24]. Alternatively, modelling can simultaneously be a sophisticated tool to improve our understanding on the relationships between environment and biota [25].

Ecological understanding is a prerequisite when it comes to selecting model environmental variables. It is plausible that traditional statistical modelling itself may not be the most rewarding way to disentangle the environmental-species relationships as it starts by assuming an appropriate data model and model parameters are then estimated from the data. By contrast, machine learning avoids starting with a data model and rather uses an algorithm to learn the relationship between the response and its predictors [26]. The novel predictive modelling technique called Boosted Regression Trees (BRT) combines the strength of machine learning and statistical modelling. BRT has no need for prior data transformation or elimination of outliers and can fit complex nonlinear relationships. The BRT also avoid overfitting the data, thereby providing very robust estimates. What is most important in the ecological perspective it automatically handles interaction effects between predictors. Due to its strong predictive performance, BRT is increasingly used in ecology [27].

As the array of available remote sensing products and statistical predictive tools is by far not fully exploited in the existing literature, the aim of the paper is to test the ecological relevance of remote sensing by combining hyperspectral remote sensing (HRS) and BRT in order to predict macrophyte and invertebrate species cover in the optically complex Baltic Sea. Specifically, we determined if HRS is sensitive to biotic patterns, and if so, how much the models can be improved by including other environmental variables that affect the species under study. We expected that HRS responds to changes in the cover of dominant species and BRT can recapture multitude of environmental-biota interactions intuitively very common in marine ecosystems. We also expected that the performance of species distribution models increases with the size of macrophyte species as bigger plants are more likely distinguishable from the surrounding environment [28]. And finally we expected that the models explain better the distribution of shallow than deep water species as water column absorbs significant amount of reflectance [29], [30].

When building models, care was taken to include ecologically the most relevant variables in order to reach the best predictions and insight on the role of various environment-biota interactions. When the selection is inadequate a model can just pick up irrelevant variables and its predictive power is low [31]. The selection of environmental variables was based on earlier results of field and experimental studies. Specifically, in the shallow waters of the Baltic Sea sediment characteristics, water exchange and exposure to waves are anticipated to shape to the largest extent benthic macrophyte and invertebrate assemblages [32]. We expect that different macrophyte and invertebrate groups have specific response functions to the studied environmental variable: e.g. seagrasses and soft bottom algae are sensitive to slight changes in wave exposure as even small increase in turbidity reduces their growth rates [33]; algae are fairly insensitive to changes in wave exposure unless hard substrate is not limited [32]; suspension feeders accumulate at elevated coastal slopes and/or exposed coasts where intense water movement provides an ample food supply [34],[35]. And we also expect large variability of species responses within each group as species are shown to have strong individualistic responses to their environment [36].

## Materials and Methods

### 1. Study area

**Figure 1:**
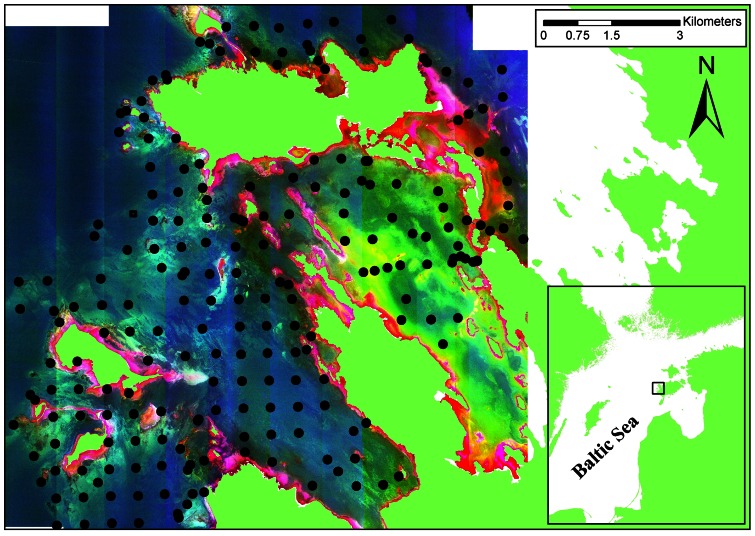
Study area. Filled dots denote the locations of the sampling stations.

The study was carried out in the Vilsandi National Park, the Western Estonian Archipelago, the Baltic Sea (Fig. 1). The study area was approximately 210 km^2^. Most of the area is less than 5 meters deep although water depth reaches 15 meters in some parts of the study area. The area is characterized by complex topography with numerous island, islets, bays, and peninsulas. The western part of the study area is wave-exposed while eastern bays are sheltered. Hard limestone substrate dominates in the exposed areas and soft silty sediments prevail in the sheltered bays. Different mixed sediments can be found in the mid-range of exposure gradient. Salinity ranges from 5 to 7.5. Regardless of low salinities, benthic flora and fauna are relatively diverse and abundant. Vascular plants (*Stuckenia pectinata*, *Ruppia maritima*, *Zostera marina*) and charophytes can be found at high densities in sheltered bays. The perennial brown alga *Fucus vesiculosus*, the red alga *Furcellaria lumbricalis* and several filamentous algae (e.g. *Ceramium tenuicorne*, *Cladophora glomerata*, *Polysiphonia fucoides*) dominate on hard substrate, occasionally giving space for the mussels *Mytilus trossulus* and the cirripeds *Amphibalanus improvisus*.

### 2. Biological sampling

A total of 207 stations were visited on August 31^st^ and September 1^st^ 2010 (Fig. 1). In order to establish the sampling stations, a grid of rectangular cells was generated with cell sizes of 50 m using the Spatial Analyst tool of ArcInfo 10 [37]. Then we calculated the values of wave exposure and inclination of coastal slopes for each grid cell (see below). The exposure and slope classes were combined to the available information on depth and bottom sediments (divided into clay, silt, sand and gravel bottoms). Using ArcInfo software sampling sites were located randomly in a way that each combination of exposure, slope, depth and sediment class had the same number of sampling sites. In areas deeper than 10 m, where the nature of bottom is homogeneous (unvegetated sands), equal distances between stations were used. At each station the seabed was sampled by deploying a remote underwater video device from an anchored boat. The camera was set at an angle of 35° below horizontal and held 1 m above the sea floor resulting in a forward view of about 2 m. A full 360° rotation was captured at each station. All recorded videos were subsequently analysed by estimating the coverage of different substrate types (mud, clay, sand, gravel, pebbles and cobbles, boulders (diameter >20 cm), rock), benthic macrophyte and invertebrate species. Biomass sampling and analysis followed the guidelines developed for the HELCOM COMBINE programme [38]. According to the Protection Rules of the Vilsandi National Park, a biological sampling does not require specific permits or approvals. The study location is not privately-owned and the study did not involve endangered or protected species.

### 3. Remote sensing

Airborne imagery was collected on September 1^st^ 2010 using hyperspectral imager CASI (Itres, Canada) belonging to the Institute for Environmental Solutions, Latvia. The spectral range of the instrument is 370−1045 nm and widths of the spectral bands are programmable. Altogether 25 spectral bands were pre-programmed in order to capture the reflectance spectra of different benthic features, to gather information about the sun glint and to provide reference data for atmospheric correction and masking land surfaces. We used the wavelength region from 480 nm to 699 nm in the model (for model description see subchapter 5 of the section of material and methods). Bands 1 to 4 (370−458 nm) were excluded from the analysis because vegetation absorbs light very strongly at these wavelengths and there is practically no difference between different benthic habitats. Besides, water absorption increases exponentially in read and near-infrared part of spectrum. A few tens of centimetres of water absorb nearly all water leaving signal at wavelengths longer than 720 nm. These spectral bands can be used to identify vegetation floating on the water surface, to estimate the amount of sun glint and to mask out land. The bands 17−25 (719−1045 nm) were used for these purposes but not in the model. The number and width of the bands were also optimized taking into account low water leaving signal and the speed of the aircraft. The aircraft was flown at an altitude of 2000 m resulting a pixel size of 1 m. A flyover was performed around midday and flight direction was chosen taking into account the sun angle in order to minimize the sun and sky glint. Flight lines were planned in the form of ellipses shifting west from the previous path. In this way, a half of the study area was flown into the sun and a half of the study area off from the sun in order to minimise the striped mosaic that may occur when flying back and forward. Pre-processing of the radiance imagery included cross-track illumination correction, geocorrection of the flight lines and mosaicking. The positional accuracy was within a range of 1 m. The longitudinal extent of the mosaicked image was 11.6 km and latitudinal extent 12.9 km.

Individual bands of the CASI image had extensive interband correlation. We used principal component analysis (PCA) on bands 5 to 16 (see previous paragraph for the explanation of why some bands were excluded from the model) for reducing the redundancy in hyperspectral images and for compressing all of the information that was contained in an original n-channel set of hyperspectral images into their principal components [39]. The spatial structure of first three principal components were highly similar to the structure of the original hyperspectral remote sensing bands; thus, the technique was highly relevant in order to reduce the redundancy in the remote sensing data. The first principal component explained more than 96% of variance and had correlation of more than 0.9 with all the CASI bands. The second component had highest correlations with bands 15 and 16. The third component correlated the most with bands 7, 8, and 9 (Table 1). All further analyses were done using three first principal components instead of original CASI bands.

**Table 1:** Correlation of the CASI bands with the PCA components.

CASI band	PC1 (96.58)	PC2 (1.43)	PC3 (0.77)
5	0.98	0.15	−0.01
6	0.99	0.12	0.05
7	0.97	−0.01	0.20
8	0.93	−0.10	0.31
9	0.90	−0.15	0.35
10	0.99	0.09	−0.01
11	0.99	0.00	−0.04
12	0.99	−0.08	−0.03
13	0.99	−0.09	−0.02
14	0.99	−0.13	−0.03
15	0.96	−0.22	0.04
16	0.97	−0.21	−0.08

The proportion of variance in the CASI bands explained by the first three principal components are shown in brackets (%).

### 4. Supporting environmental variables

Based on a bathymetry raster of 50 m pixel resolution (available at the Estonian Marine Institute, University of Tartu) the inclination of coastal slopes was calculated using the Spatial Analyst tool of ArcInfo software [37]. High values of coastal slopes indicate the occurrence of topographic depressions or humps at the measured spatial scale. Low values refer to flat bottoms.

The Simplified Wave Model method was used to calculate the wave exposure for mean wind conditions represented by the ten-year period between 1 January 1997 and 31 December 2006 [40]. A nested-grids technique was used to take into account long-distance effects on the local wave exposure regime. The resulting grids had a resolution of 25 m. In the modelling the shoreline was divided into suitable calculation areas, fetch and wave exposure grids were calculated and subsequently the separate grids were integrated into a seamless description of wave exposure along the study area. This method results in a pattern where the fetch values are smoothed out to the sides, and around island and skerries in a similar way that refraction and diffraction make waves deflect around islands.

### 5. Modelling methods

The contribution of different environmental variables on the coverages on benthic species was explored using the Boosted Regression Tree technique (BRT) and the BRT models were used to predict the species coverages. BRT models are capable of handling different types of predictor variables and their predictive performance is superior to most traditional modelling methods. The BRT iteratively develop a large ensemble of small regression trees constructed from random subsets of the data. Each successive tree predicts the residuals from the previous tree to gradually boost the predictive performance of the overall model. Although BRT models are complex, they can be summarized in ways that give powerful ecological insight [27].

**Figure 2:**
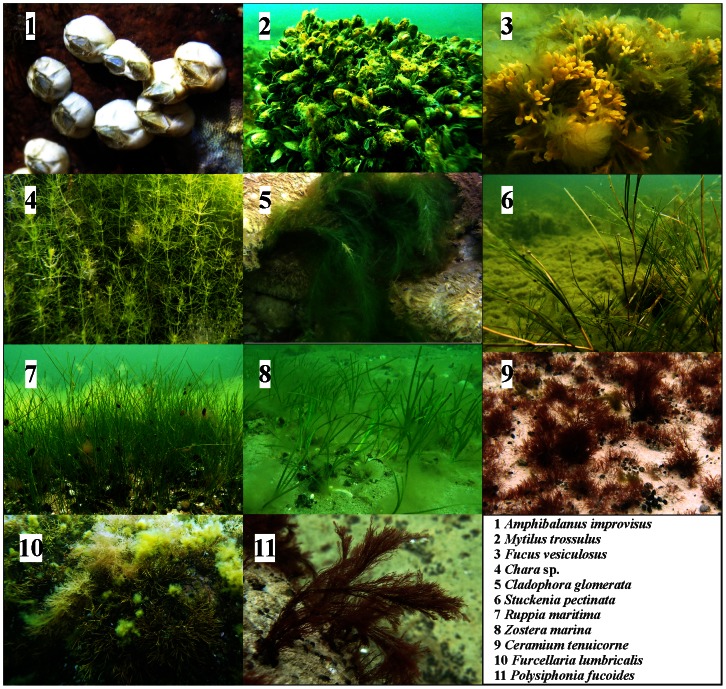
Still photographs of benthic species in the study area.

Prior to using the BRT models, we stored our data in a data.frame object, which is a data structure used by the R software. A data frame may be regarded as a matrix with columns of differing modes and attributes. It may be displayed in matrix form, and its rows and columns extracted using matrix indexing conventions. The names of the variables must match exactly the names used in the gbm function calls. For BRT modelling the independent variables were: depth (primarily a proxy for light), sediment characteristics (cover of hard bottoms), exposure to waves (proxy for wave disturbance), coastal slope (proxy for water exchange) and three principal components of CASI bands. They were regressed to predict the coverage of benthic macrophyte and invertebrate species. As organism size and morphology is expected to determine its interactions with the ambient environment [41], test organisms have been selected to cover small and large seagrass, algae with fine and coarse thalli and sessile benthic invertebrates. Considering that algal colour can interfere with CASI measurements, green, brown and red algae were included into the study (Fig. 2).

**Figure 3:**
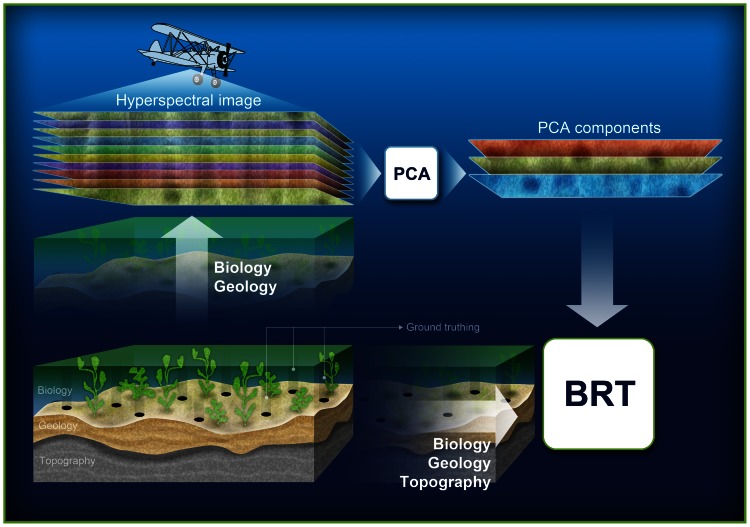
Schematic flowchart on the main methodological steps used to combine hyperspectral remote sensing and Boosted Regression Tree technique in order to predict macrophyte and invertebrate species cover in the optically complex Baltic Sea.

In fitting a BRT the learning rate and the tree complexity must be specified. The learning rate determines the contribution of each successive tree to the final model, as it proceeds through the iterations. The tree complexity fixes whether only main effects (tree complexity  = 1) or interactions are also included (tree complexity >1). Ultimately, the learning rate and tree complexity combined determine the total number of trees in the final model. For each species, multiple models were run varying either the model learning rate (between 0.01 and 0.0001), number of trees (between 1000 and 10,000), and number of splits (1 and 5). Then the optimum model was selected based on model performance. Typically, optimal learning rates, number of trees and interaction depth were 0.001, 3000 and 5, respectively. Model performance was evaluated using the cross validation statistics calculated during model fitting [42]. A random 20% of the data was assigned for testing model accuracy. As a total of 207 stations were visited, all these samples were used in the modelling i.e. n = 207 for each species. All statistical analyses were done in the statistical software R version 2.0.1 using the gbm package [43]. We used the BRT script provided by [27]. All major methodological steps from field sampling to the BRT modelling are summarized in the schematic flowchart on Figure 3.

In addition, we compared the performance of BRT modelling with a common supervised classification technique, the Spectral Angle Mapper (SAM) [44] in order to demonstrate a significant methodological improvement. SAM is a spectral classification scheme that uses an n-dimensional angle to match pixels to reference spectra. We chose the reference spectra from the imagery based on the results of field sampling. A reflectance of half of the sampling points for each class was used as reference spectra and the rest were used for validation. It is possible to use different angles in SAM. Smaller angle means tighter fit between the reference and image spectra. We used variable angles in SAM as using too small angle results in most of the image being unclassified. The criterion for selecting the angle was to classify the full image. Due to the differences in approaches, the BRT models and supervised classification are not directly comparable as the BRT model provides information on both species identity and quantity whereas the supervised classification technique informs us on species presences only. Therefore, we also analysed, using the one-way ANOVA, whether the cover of macroalgae differs among supervised classification classes (classification levels: species present, species absent).

## Results

### 1. Object features contributing to remote sensing

The BRT models identified that success at object detection depended largely on its properties and was uncoupled of its environment. Specifically, hyperspectral remote sensing when combined with the BRT modelling described a significant proportion of variability in the cover of benthic macroalgae and invertebrates but not higher plants including seagrasses (Table 2; Fig. S1). On the other hand, a traditional supervised classification technique only performed slightly better than chance as indicated by moderate class agreement values. Moreover, when we analysed whether the cover of macroalgae differs among supervised classification classes (classification levels: species present, species absent) then we realized that in majority of cases, the differences were not statistically significant (Table 2).

**Table 2:** The percentage of variance explained by the BRT species models including only remote sensing variables (Remote model total) and combining both remote sensing and abiotic environmental variables (Combined model total).

Species	Remote model total	Combined model total	Depth	Exposure	Slope	Sediment	Remote sen sing variables	Class agree ment	P
*Amphibalanus improvisus*	35	48	21	21	8	22	28	NA	NA
*Mytilus trossulus*	39	61	9	39	15	15	22	NA	NA
*Fucus vesiculosus*	43	76	12	16	9	25	38	53	0.354
*Chara* sp.	69	77	4	63	1	6	26	66	0.011
*Cladophora glomerata*	61	77	6	42	2	8	42	69	0.743
*Stuckenia pectinata*	25	71	15	24	4	33	24	62	0.154
*Ruppia maritima*	11	32	10	19	27	11	33	64	0.180
*Zostera marina*	16	58	26	17	8	32	17	64	0.346
*Ceramium tenuicorne*	13	60	19	21	23	21	16	53	0.160
*Furcellaria lumbricalis*	63	71	46	9	3	2	40	82	0.015
*Polysiphonia fucoides*	42	55	28	10	5	9	48	60	0.188

For the models combining remote sensing and abiotic environmental variables, the separate explained deviance of abiotic environmental and remote sensing variables are shown (% of combined model total). In addition the column “class agreement” denotes the percentage of match between predicted and observed species presences using a common supervised classification technique. Higher than 50% class agreement indicates that supervised classification is performing better than chance. NA refers that the supervised classification technique was not able to classify the image. Please note that the shown percentage agreements of BRT models and supervised classification are not directly comparable as the BRT models provide information on both species identity and quantity whereas the supervised classification technique only informs us on species presences. Please also note that if probability “P” value is less than 0.05 then differences in species cover among supervised classification classes (levels: species present, species not present) are actually statistically significant.

**Figure 4:**
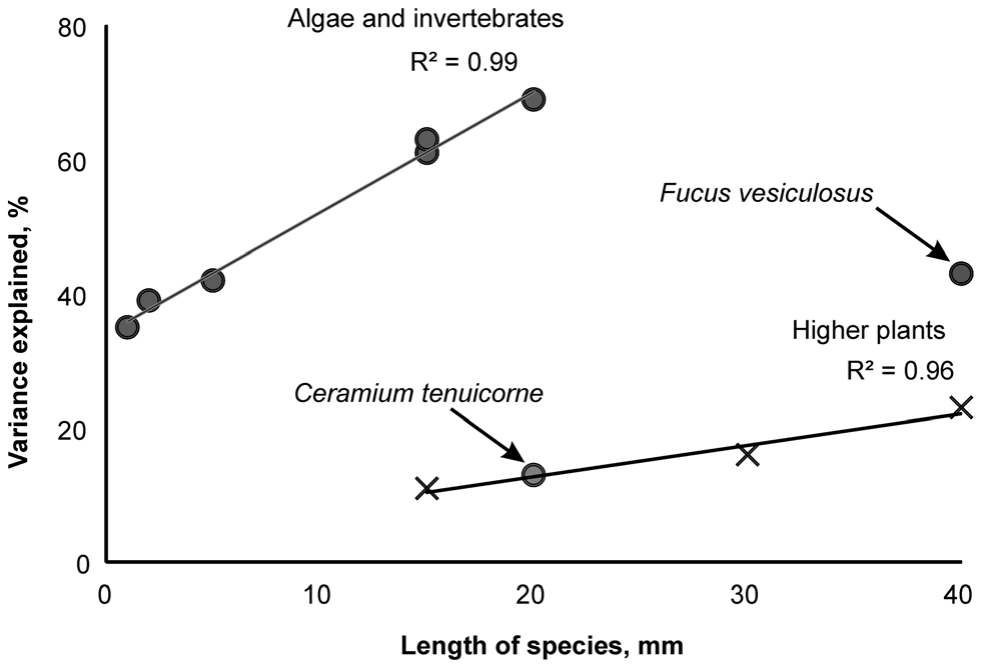
Relationship between object size and predictive power of remote sensing variables in the BRT models. Each data point represents single species. Filled circles denote macroalgae and benthic invertebrates whereas crosses denote higher plants.

As expected there was a strong linear relationship between the size of organism and the predictive power of remote sensing variables in the BRT models. However, the macroalgae-invertebrate group had a significantly higher regression slope than higher plants (Fig. 4). There were also two outliers of these linear relationships. In terms of predictive power, the red algae *C. tenuicorne* fitted to the regression line of higher plants and the cover of brown alga *F. vesiculosus* was about 2.5 times less predictable as expected from its size.

Contrary to our expectations, there was no significant relationship between the average depth inhabited by species and the variance explained by remote sensing variables. The highest performing boosted regression tree models (including only remote sensing variables) were for the shallow-water green algae *C. glomerata* (r^2^ = 0.61) and *Chara sp*. (r^2^ = 0.69) but, surprisingly, also the deep-water red alga *F. lumbricalis* (r^2^ = 0.63).

In general, broad taxonomic category did not explain how well remote sensing variables contributed to the BRT models. Only the cover of green algae had a very strong signal in the remote sensing variables. The models of red algae and higher plants had very high within group variability in their predictive power.

### 2. Effect of abiotic environment on species

When remote sensing data were combined with coastal geomorphology and weather data then the performance of BRT models were significantly improved with the half of species exceeding model r^2^ values above 0.70 (Table 2). Specifically, the higher plants, the brown alga *F. vesiculosus* and the bivalve *M. trossulus* models benefitted the most from combining remote sensing and other environmental variables. In contrast, the models of green algae were practically not enhanced.

The BRT modelling showed that the effect of environmental variables on the patterns of species distribution largely varied among the studied species. However, some generalities can be drawn. Besides remote sensing variables, exposure and partly water depth and sediment characteristics were the best predictors for the majority of the BRT models (Fig. S2). Overall, wind patterns best explained variability in the coverage of shallow water species whereas coastal geomorphology largely contributed to the models of deep water species. All species inhabiting the shallowest part of the sea were highly sensitive to slight changes in exposure levels with their cover exponentially decreasing with increasing wave activity. Deeper water species, including higher plants, had various responses to exposure and in general, the responses were small in magnitude. Among deeper water species only *R. maritima* inhabited a relatively narrow exposure range.

Our data also revealed that diverse functional relationships also exist between availability of hard substrate and species cover. An increased availability of hard substrate linearly raised the cover of suspension feeders, *F. vesiculosus* and *P. fucoides* and decreased the cover of *Z. marina* over an entire sediment gradient. *R. maritima* benefitted the increment in the share of soft sediment containing up to 40% sand grains. Other species avoided mixed sediments and primarily inhabited either truly hard (*C. tenuicorne*) or soft bottom habitats (*S. pectinata*). And finally there was a group of species that were practically insensitive to change in sediment characteristics (*F. lumbricalis*, *Chara* sp., *C. glomerata*).

As expected exposure to waves was the key correlate of the cover of suspension feeders and the relationship approximated a logistic function. From low to mid range exposure level the cover of both *M. trossulus* and *A. improvisus* was almost insensitive to change in exposure. At mid range of exposure the elevated wave activity exponentially increased the cover of suspension feeders until a certain threshold was reached and beyond that point other variables controlled the populations of suspension feeders in the model. The models also showed a clear niche separation of these benthic taxa with *M. trossulus* inhabiting steeply sloping shores and *A. improvisus* gently sloping shores.

**Figure 5:**
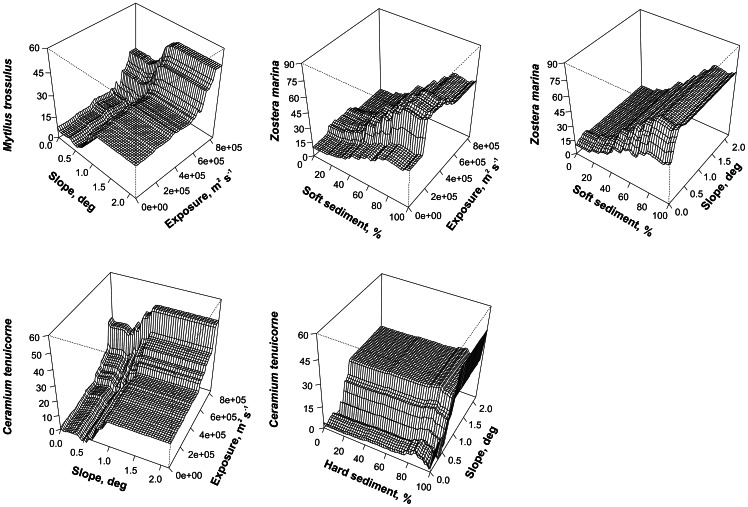
Selected interactions between abiotic environmental variables and species cover. Environmental variables are as follows: exposure – exposure to waves, slope – inclination of coastal slope, soft sediment – percentage cover of soft substrate, hard sediment – percentage cover of hard substrate.

Besides suspension feeders coastal slope also contributed to the cover of *R. maritima* and *C. tenuicorne* with elevated slope values increasing the species cover. Other macrophyte species were insensitive to changes in coastal slope. However, when combined with other variables (e.g. sediment characteristics) coastal slope interactively contributed to the cover of *Z. marina*. All other interactions did not differ in direction of effect from the separate influence of environmental variables on species cover (Fig. 5).

## Discussion

This study demonstrated the strength of combining machine learning, statistical modelling, remote sensing and traditional spatial modelling variables in order to model the species distribution of marine benthic macrophyte and invertebrate species. Even though the water column absorbs a significant amount of water leaving signal [29], [30], [45] and the strength of correlation between remote sensing variables and biotic patterns is expected to be higher in terrestrial environments than in aquatic environments, our models reached or even exceeded the predictive power of terrestrial models. If the terrestrial models often describe 50−75% of variability in biotic patterns [46], [47] then the aquatic models rarely reach such predictive power, often explaining only up to 40% of variability [48] but see [20] for higher predictive power using non-boosted regression trees. As such, our modelling approach performed far better than the traditional methods. Considering the optical complexity of the Baltic Sea compared to open ocean environments [49], [50], the results indicate a strong potential of the method in the modelling of aquatic species in the large variety of ecosystems.

The same CASI imagery and field sampling data were used in another remote sensing study [51]. This study demonstrated that a conventional supervised classification technique could not separate many of the benthic habitats from each other. The finest classification scheme achieved contained only 8 broad classes (bare substrate, brown algae, red algae, dense higher plants, etc.) and optically deepwater class. This is because the optical signatures of species were not different for any remote sensing sensors. Some of the species either covered too small area of 1 m^2^ pixel or were growing under larger vegetation. Consequently, these species could not be mapped with remote sensing as they did not contribute to the optical signal remote sensing instruments were measuring. Thus, the level of detail provided by the supervised classification technique and BRT modelling is not comparable. Moreover, the BRT models provide information on species cover and therefore carry much more information compared to the majority of previous models that just predicts species distributions.

### 1. Object features contributing to remote sensing

Remote sensing varied in its effectiveness to explain the cover of different benthic macrophyte and invertebrate species. The expectation that the role of remote sensing variables in the species distribution models increases with the size of macrophyte and invertebrate species was confirmed. In fact the importance of image object size in mapping has been emphasized both in terrestrial and aquatic environments [52], [53], [54]. This is because with the increasing object size the probability that objects are omitted and/or wrongly detected substantially decreases, thus the prediction accuracy substantially increases. However, studies that specifically target prediction accuracy related to object size are almost lacking and the focus is almost strictly on the issues of image classification [55].

Organism size also reflects the physiological state associated with the allometric relationship between size and metabolic rate of the organisms [56]. In this respect remote sensing better detects physiologically less active functional forms e.g. brown and red perennial algae compared to small ephemeral seaweeds. A plausible biophysical mechanism for the observed effect is the presence of a protective (i.e. remotely well detected) tissue associated with the perennial algae. Besides, independently from the size of an object, the functional form of organisms seems to determine how well the species is detected. Namely, our study clearly showed that the relationship between object size and its prediction significantly differed among higher plants and other marine organisms. It is plausible, though, that habitats characterized by higher plant species are in general more turbid than areas inhabited by hard bottom macroalgae and sessile invertebrates [32] and therefore the observed differences in the model's predictive power may partly arise from water properties and not on colour, texture and shape of an object. However, this is not the only explanation for the results. As an example charophytes inhabit sandy/silty bottoms and in such habitats wind induced resuspension of fine particles is very likely; nevertheless, they were the best predicted objects in our study.

There were also two outliers of the observed relationships. Remote sensing method was far less sensitive to the detection of the brown seaweed *F. vesiculosus* than was predicted from the size of seaweed. This is exactly the opposite of what we expected considering that among the studied species *F. vesiculosus* encompasses the largest gradients of environmental variability and occurs at high frequency [32] both facilitating the emergence of strong relationships between remote sensing variables and the brown seaweed cover. Nevertheless, *F. vesiculosus* hosts a wide array of epiphytic algae and invertebrates [57] and the heavy epiphyte load may haze its optical signature and thus hinder the species detection. The prevailing epiphytic algae *Pilayella littoralis* is not host specific in the study area. Besides *F. vesiculosus*, *P. littoralis* may grow on other perennial macroalgae, directly attach to hard substrate or even form a drifting algal mats [58], thus making the separation of *F. vesiculosus* habitat very difficult in terms of their optical properties from e.g. other perennial macroalgal and/or drifting algal habitats. Another exception includes the detection of the red alga *C. tenuicorne*. Although, the species inhabits shallow water environments and therefore can be potentially well detected, the red alga has a translucent appearance and is difficult to be seen. Moreover, as the red alga do not appear to tolerate high irradiance it often forms an understory of other macroalgae [59] which further complicates its detection from sea surface.

Surprisingly, there was little or no difference how remote sensing detected shallow and deep water species. Green algae, which grow in the shallowest parts of the study area, had a very strong signal in the remote sensing variables. Similarly, the highest performing model was also for the deep-water red alga *F. lumbricalis*. In general, the predictive power of models of red algae and higher plants were independent of depth. It is plausible that a lack of depth dependency reflects large spatial differences in water transparency in our study area. Specifically, soft bottom substrates tend to be systematically more turbid than hard bottom habitats and thus, the detection of hard bottom macrophytes is expected to be more efficient compared to soft bottom macrophytes.

### 2. Effect of abiotic environment on species

Accurate prediction and explanation are fundamental objectives of statistical analysis and BRT attains both of these objectives [60]. By doing so, they can determine relationships between the response and the predictors, and thus they have a high potential to explain the underlying processes behind the pattern of species distribution in the seascape. In contrast, traditional statistical models such as linear regression analyses are routinely used to explain data relationships, but despite its simplicity and ease of use, they are often relatively poor predictors. As expected our models easily predicted a large quantity of macroalgal and invertebrate species cover and recaptured multitude of interactions between environment and biota, contrasting earlier results on the ease of use of remote sensing methods in marine environments [30], [61].

The studied species varied widely in how they responded to the environment. It is a well-publicized fact that species' traits determine the strength and direction of relationship between environment and biota [62]. Specifically, some species have wide tolerance ranges and are found over over a wide range of habitats. However, other species have very narrow tolerance ranges and are therefore very limited in their ranges. The BRT models clearly distinguished between such specialist and generalist species. The specialist species were characterized by a narrow peak in the the functional form of a relationship between environment and species cover, the peak indicating the optimum range of species natural distribution. The commonest examples of specialists were the charophytes and the higher plants *Stuckenia pectinata* and *Zostera marina*. All these species hold a very specific biological niche in the coastal ecosystem i.e. *Chara* sp. preferred shallow depths and very sheltered areas, *S. pectinata* inhabited fine sediments in shallow and sheltered areas and *Z. marina* preferred moderate depths and moderate exposure regimes and avoided flat bottoms. The gereralist species such as the cirriped *A. improvisus* and the brown alga *F. vesiculosus* had high cover over values over a wide range of environmental conditions.

The BRT models also identified the most important environmental variables limiting the spread of the studied species in the study area (i.e. those environmental variables having the highest contribution to the model performance). Specifically, our models predicted strong relationship between wave patterns, benthic macrophyte and invertebrate cover in the shallowest parts of the sea but not deeper down. This conforms to the earlier evidences that in the dynamic coastal habitats local weather patterns largely define the observed biotic patterns [63], [64]. In our study area such effects are related mainly to the duration of ice cover and are probably due to the varying intensities of ice abrasion [32]. Strong physical disturbance in shallow exposed areas may even counteract the effects of nutrient loading as ice abrasion periodically removes the excess biomass i.e. attached macrophytes and sessile invertebrates. Deeper down the role of mechanical disturbance is reduced and the benthic macrophyte species are controlled by the availability of substrate, nutrient and light and biotic interactions [12], [32].

Wave exposure and resulting sediment patterns were seemingly major controls of the distribution of higher plants, with the mosaic of sediment supporting high species richness and variability in benthic communities in the study area [65]. There are, plausibly, several physical mechanisms behind the observed relationship. Firstly, the availability of soft substrate is prerequisite for the establishment of the species. Secondly, sediment modulates the flow above seabed [66], [67] and the intensity of flows is directly related to the cover pattern of the macrophytes [68], [69]. In soft sediments, water flow also determines the light climate; i.e., large waves may cause considerable resuspension of sediments and prolonged periods of poor light conditions [69]. Thirdly, small scale topographic heterogeneity i.e. boulder fields may provide the species refuges against physical disturbances including ice scouring and mechanical stress due to waves [70], [71].

In addition, the BRT modelling indicated that seagrass and similar group of plants were poorly predicted by our models. If the ephemeral species such as *C. glomerata* and *C. tenuicorne* are very responsive the environment over short time intervals and be very influenced by local conditions [72] then seagrasses are known to modify their local abiotic environment by trapping and stabilizing suspended sediments and thereby improving water clarity and seagrass growth conditions [73]. Thus, seagrass distribution is expected to be less coupled with their adjacent abiotic environment compared to many non-engineering species. Moreover, the cover of seagrass species is rather a function of a colonization history that spans decades to centuries [74].

The universal relationship between wave climate and the cover of suspension feeders suggest that suspension feeders are food limited in the study area. Besides, it is expected that suspension feeders benefit from the increased water flow on the more complex bottom topography, as a rising flow velocity improves their food supply, and positive interactive effects between current velocity and phytoplankton biomass are expected [75], [35].

In addition to the direct effect of food transport, the relationship between wave exposure and cover of suspension feeders may involve indirect interactions between macroalgae and suspension feeders. Namely, macroalgae are known to outcompete benthic suspension feeders at shallow depths, and lush macrophyte communities are therefore often characterised by low densities of suspension feeders [76]. Moderate exposure to waves and ice disturbance partly removes the algal carpet, thus releasing benthic suspension feeders from such interspecific competition [77]. Too great an ice disturbance, however, also removes sensitive suspension feeders. This may explain why *A. improvisus* inhabits gently sloping shores where such mechanical disturbance is not as severe as in steeply sloping shores.

As seen in some examples above, the BRT modelling enabled to identify critical thresholds marking tipping points where even a slight change in environmental conditions resulted in the abrupt shifts of species distribution. Understanding factors that shape niche width, species coexistence and thereby habitat diversity are of utmost importance in ecology both theoretically and for conservation policies. Such knowledge can be potentially used to predict species distribution e.g. under current environmental conditions and the projected influences of different management strategies and climate change scenarios. Therefore, our models can be seen as a valuable tool for improving environmental protection of coastal benthic habitats.

## Conclusions

The machine learning technique combined with statistical modelling, remote sensing and traditional spatial modelling variables succeeded in identifying, constructing and testing functionality of abiotic environmental predictors on the coverage of benthic macrophyte and invertebrate species. Although correlative in nature, the resulting response curves matched well with the current understanding on the interdependence of abiotic environment and benthic species. The models also provided many ecologically realistic second-order interactions that can be tested in controlled experimental conditions. However, our study showed that the majority of species had individualistic responses to their environment. This provides the strong conceptual argument for modelling individual species rather than communities and fosters the usage of machine learning over traditional modelling methods in order to unravel the environment-biota interactions.

Certainly, the present study has some limitations that need to be taken into account when considering the study and its contributions. Some of these limitations, however, can be seen as fruitful avenues for future research under the same theme. Clearly, the size of the object affects its detection together with aspects such as water transparency. Nevertheless, we believe that further development of remote sensing instruments and signal processing technology ensure stable detection of small objects. It must be stressed that only objects, that are situated at the top of biota, are seen by remote sensing. Thus, the patterns of multi-layered communities are currently relied by spatial modelling component only. A single case study naturally brings forth many limitations as far as the generalisation of the results of the study is concerned. Thus, it becomes rewarding to seek generic standardized procedure to map multiple species in multiple areas. Such maps would greatly expand our capacity to understand biotic patterns, their changes and causes and thereby improve ecological theory and potentially preserve endangered seascapes for future generations.

## Supporting Information

Figure S1 **Functional relationships between remote sensing variables and species cover.** PC 1, PC 2 and PC 3 are the first principal components of CASI bands. Percentage shows the separate contribution of remote sensing variables in the model.
(TIF)

Figure S2 **Functional relationships between studied environmental variables and species cover.** Environmental variables are as follows: depth – water depth, exposure – exposure to waves, slope – inclination of coastal slope, sediment – percentage cover of soft substrate. Percentage in each plot shows the separate contribution of environmental variables in the model.
(TIF)
